# Adenocarcinoma developed from remnant cystic duct after cholecystectomy

**DOI:** 10.1186/1471-230X-14-175

**Published:** 2014-10-06

**Authors:** Jae Hyuk Do, Yoo Shin Choi, Eun Young Ze

**Affiliations:** Department of Internal Medicine, Chung-Ang University Hospital, 224-1, Heuk Seok-Dong, Dongjak-Ku, Seoul, 156-755 South Korea; Department of Surgery, Chung-Ang University Hospital, 224-1, Heuk Seok-Dong, Dongjak-Ku, Seoul, 156-755 South Korea

**Keywords:** Remnant cystic duct, Adenocarcinoma, Postcholecystectomy

## Abstract

**Background:**

Cystic duct adenocarcinoma is a rare disease because the cystic duct has a short length and a narrow cavity. Cystic duct adenocarcinoma accounts for 8% of all gallbladder adenocarcinoma; of these, adenocarcinoma that develops from the remnant cystic duct after cholecystectomy is extremely rare. We report a case of incidentally detected adenocarcinoma that developed from the remnant cystic duct in a patient with history of cholecystectomy.

**Case presentation:**

A 74-year-old Korean woman visited our hospital with abdominal pain. Her past medical history included cholecystectomy for acute cholecystitis with gallstones 10 years previously. Imaging of the abdomen demonstrated inflammation of the remnant cystic duct with multiple impacted stones. Complete removal of the remnant cystic duct with stones was performed. The pathologic report showed severe inflammation with abscess formation and an unexpected adenocarcinoma that appeared to invade the perimuscular connective tissue. The second operation (confirmation of the resection margin of the remnant cystic duct, wedge resection of the liver, and lymphadenectomy) was performed due to suspicion of pT2. There were no cancer cells in the resection margin of the remnant cystic duct, liver, or lymph nodes (0/6). The final histopathological diagnosis was pT2N0M0. She recovered without any complications. The patient is still living 1 year after surgery without recurrence or metastasis.

**Conclusions:**

We report a rare case of adenocarcinoma that developed from the remnant cystic duct in a patient who underwent cholecystectomy.

## Background

Cystic duct adenocarcinoma is a rare disease because the cystic duct is anatomically short in length and has a narrow cavity [1, 2]. When the narrow lumen of the cystic duct becomes obstructed, symptoms such as abdominal pain or jaundice may develop early and the prognosis may be quite favorable; however, later on, the tumor seems to behave like bile duct carcinoma [3]. The remnant cystic duct (RCD) is not protected from carcinoma, although this is an extremely rare occurrence compared to malignancy of the intact cystic duct. We report a very rare case of incidentally detected adenocarcinoma that developed from the RCD.

## Case presentation

A 74-year-old Korean woman was admitted to our hospital with right upper quadrant (RUQ) abdominal pain, nausea, and vomiting which began the preceding day. She had no smoking history or significant family history. Her past medical history included cholecystectomy for acute cholecystitis with gallbladder (GB) stones 10 years prior. At that time, the pathologic report was inflammation of the GB. Two years before admission, a computer tomography (CT) scan of the abdomen performed for routine screening revealed two small stones (4 mm, 3 mm) in the RCD, and two small stones (3 mm, 2 mm) in the distal common bile duct (CBD). Endoscopic retrograde cholangiopancreatoscopy (ERCP) was performed, and the two CBD stones were removed. At that time, surgery was recommended for the RCD stones, but the patient refused the operation.

**Figure 1:**
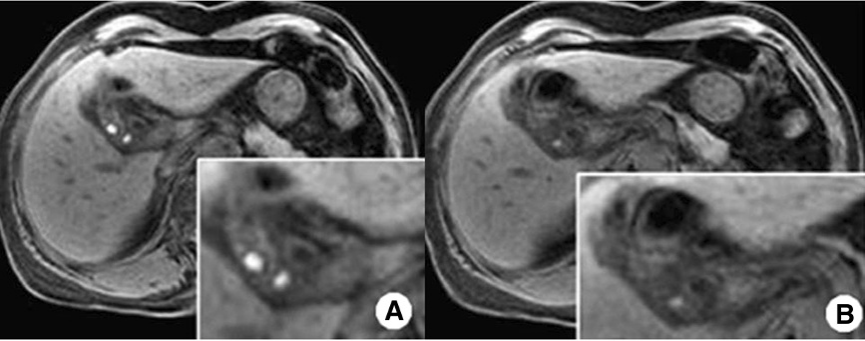
**Findings on magnetic resonance cholangiopancreatography. A**: Inflammation in the remnant cystic duct with multiple impacted stones; **B**: Focal enhancing of wall thickening in the remnant cystic duct adjacent to the common bile duct.

**Figure 2:**
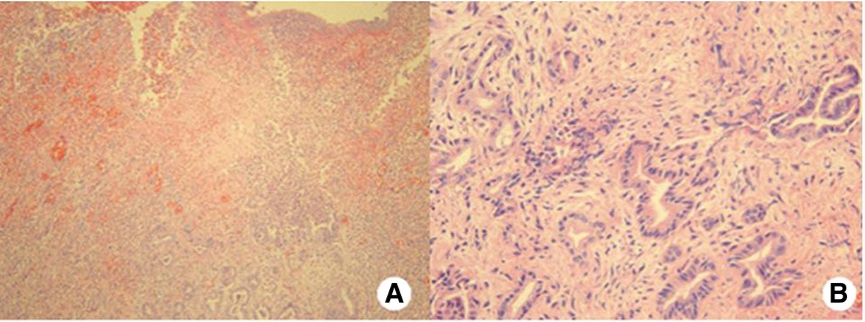
**Microscopic findings. A**: Severe inflammation with atypical glands proliferation (Hematoxylin-eosin staining, original magnification ×100). **B**: Atypical glands with invasive pattern, indicating adenocarcinoma (Hematoxylin-eosin staining, original magnification ×400).

At admission, she was afebrile and physical examination showed tenderness of the RUQ of the abdomen. Blood test and laboratory data indicated an elevated white blood cell count (10360 μl^-1^; normal < 9000 μl^-1^) and elevated serum concentrations of high sensitivity C reactive protein (22.98 mg/L; normal < 1.0 mg/L). However, the serum concentrations of total bilirubin, alanine aminotransferase, and alkaline phosphate, as well as tumor markers, including carcinoembryonic antigen and carbohydrate antigen 19–9 were within the respective normal ranges. Both CT scan and magnetic resonance imaging of the abdomen demonstrated inflammation of the RCD with multiple impacted stones (Figure 1).Complete removal of the RCD was performed with a preoperative diagnosis of inflammation of the RCD and multiple impacted stones. The size of the excised RCD was 2 cm × 1 cm and the wall was thick. There were multiple stones (1 cm × 1, 2 ~ 3 mm × 5) and pus-like bile. The pathologic report indicated chronic active inflammation with abscess formation and adenocarcinoma that appeared to invade the perimuscular connective tissue (Figure 2). On positron emission tomography CT scan, there was no evidence of metastasis.

The second operation was planned due to suspicion of pT2. Confirmation of the resection margin of the RCD, wedge resection (segment 4b and 5) of the liver, and lymphadenectomy were performed. There were no cancer cells in the resection margin of the RCD, liver, or lymph nodes (0/6). The final histopathological diagnosis was pT2N0M0. The patient recovered without any complications, and is still living 1 year after surgery without any recurrence or metastasis.

## Discussion

The normal cystic duct connects the GB to the common hepatic duct to form the CBD, and it is considered the most important structure to be identified during cholecystectomy. The normal cystic duct usually measures 4 to 6 cm in length, and 1 to 5 mm in diameter.

During cholecystectomy, a variable length of cystic duct is left as a remnant. The length of the normal cystic duct measures 5 to 25 mm and the caliber is approximately 4 mm [4]. If the RCD is longer, RCD syndrome occurs more frequently and long cystic duct stump can be a site where gallstones can be formed “de novo” [5]. On the contrary, if the RCD is shorter, a benign CBD stricture is more likely to occur. A RCD measuring longer than 1 cm is called a cystic duct remnant [6].

Cystic duct adenocarcinoma is a rare entity, and is considered a GB cancer [7]. Although the risk factors of GB cancer are being reported, those of cystic duct adenocarcinoma were reported nil [8]. Cystic duct adenocarcinoma is reported that it accounts for 8% of the entire GB cancer [2]. The diagnosis of cystic duct adenocarcinoma distinguishes primary cystic duct adenocarcinoma from secondary cystic duct adenocarcinoma metastasized from GB cancer or cholangiocarcinoma. In 1951, Farrar et al. first described the diagnostic criteria for cystic duct adenocarcinoma that included (a) growth restricted to the cystic duct, (b) absence of neoplastic process in the GB, hepatic, or CBD, and (c) histological evidence of adenocarcinoma. According to these criteria, primary cystic duct adenocarcinoma accounts for 2.6 to 12.6% of extrahepatic bile duct cancer and 1.5% of GB cancer [7, 9]. In 2003, Ozden et al. revised these criteria and extended them in order to include advanced cases. The new working definition of cystic duct adenocarcinoma describes cystic duct adenocarcinoma as a GB tumor whose center is located in the cystic duct [10]. RCD cancer is now defined as a cystic duct adenocarcinoma occurring >5 years post-cholecystectomy [2]. In the described case, the diagnosis matched both Farrar’s and Ozden’s criteria.

Symptoms may develop earlier in cystic duct adenocarcinoma than in GB cancer. The anatomical structure of cystic duct adenocarcinoma can lead to cystic duct obstruction, which causes symptoms like abdominal pain, jaundice, palpable mass due to GB distension, and cholecystitis with fever, chills and other systemic inflammation. These symptoms may aid in the early detection of cystic duct adenocarcinoma [11]. Accordingly, early detection is the reason why bile duct invasion, lymph node metastasis, and distant metastasis are relatively rare in cystic duct adenocarcinoma [7, 12]. Similarly, the resectability rate in cystic duct adenocarcinoma was reported to be close to 100%. On the other hand, the resectability rate in extrahepatic bile duct or GB cancer is less than 30%. This high resectability rate in cystic duct adenocarcinoma has improved the prognosis. The median survival period of cystic duct adenocarcinoma is longer (20.2 months) than extrahepatic bile duct (3.2-11.4 months) or GB cancer (5.8 months) [10].

However, as in the present case, patients who have undergone cholecystectomy have late onset of symptoms and early diagnosis may be difficult. Moreover, in this case, the patient was advised to have removal of RCD for incidentally detected RCD stones two years prior, but she declined the surgery at that time. In advanced cystic duct adenocarcinoma or cystic duct adenocarcinoma with lymph node metastasis, there is no chance of a radical resection and the prognosis is poor. Therefore, early diagnosis is very important [7].

As in this case, there have been more cases of pathologic diagnosis at the time of surgery or after surgery rather than preoperative diagnosis. However, with the development of diagnostic methods such as ERCP, magnetic resonance cholangiopancreatography, endoscopic ultrasound, and intraductal ultrasound, more cases of preoperative diagnosis have been reported recently [10]. Moreover, the evaluation of the extent of tumor infiltration through the bile duct wall prior the operation is mandatory for planning curative surgery.

Although the lymph nodes and bile duct are not usually involved in adenocarcinoma of the cystic duct, cholecystectomy usually leads to an unsatisfactory outcome, possibly due to bile duct invasion and perineural invasion. Thus, the combined resection of the GB and bile duct with lymph node dissection is the choice of treatment for adenocarcinoma of the cystic duct, offering a better prognosis and higher survival rate, including a higher chance of potential cure [12, 13].

Despite the lack of randomized trials, many retrospective articles have shown that postoperative adjuvant radiation therapy is effective in increasing the overall survival in patients with GB or extrahepatic bile duct adenocarcinoma, particularly those with positive surgical margins [14–16]. We therefore consider postoperative adjuvant radiation therapy in cases of advanced cystic duct adenocarcinoma with positive surgical margins, but more studies are needed to establish its efficacy.

## Conclusion

In conclusion, we report a case of adenocarcinoma that developed from the RCD in a patient who underwent cholecystectomy 10 years ago. RCD cancer is extremely rare; complete removal of the RCD and a second operation (confirmation of the resection margin of the RCD, wedge resection of the liver, and lymphadenectomy) were successfully performed under the suspicion of pT2. The patient is still living 1 year after surgery without any recurrence or metastasis. Finally, according to literature data, the cystic duct stump should be made as short as possible without impinging on the CBD in order to avoid developing complications related to stasis of bile in the retained cystic duct.

## Consent

Written informed consent was obtained from the patient for publication of this case report and any accompanying images. A copy of the written consent is available for review by the Editor of this journal.

## Abbreviations

RCD: Remnant cystic duct
RUQ: Right upper quadrant
GB: Gallbladder
CT: Computer tomography
CBD: Common bile duct
ERCP: Endoscopic retrograde cholangiography.
